# Nanogels Derived from Fish Gelatin: Application to Drug Delivery System

**DOI:** 10.3390/md17040246

**Published:** 2019-04-25

**Authors:** Min Gyeong Kang, Min Young Lee, Jae Min Cha, Jung Ki Lee, Sang Cheon Lee, Jeehye Kim, Yu-Shik Hwang, Hojae Bae

**Affiliations:** 1 Department of Bioindustrial Technologies, College of Animal Bioscience and Technology, Konkuk University, Seoul 05029, Korea; vq1004pv@naver.com (M.G.K.); ljk7725@naver.com (J.K.L.); mari_88@naver.com (J.K.); 2 Smart Healthcare Medical Device Research Center, Samsung Medical Center, 81, Irwon-ro, Gangnam-gu, Seoul 06351, Korea; mylove213@daum.net; 3 Department of Medical Device Management and Research, Samsung Advanced Institute for Health Sciences & Technology, Sungkyunkwan University, 81, Irwon-ro, Gangnam-gu, Seoul 06351, Korea; 4 Department of Mechatronics, College of Engineering, Incheon National University, Incheon 22012, Korea; jae.m.cha@gmail.com; 5 Department of Maxillofacial Biomedical Engineering and Institute of Oral Biology, School of Dentistry, Kyung Hee University, Seoul 02447, Korea; schlee@khu.ac.kr; 6 Department of Stem Cell and Regenerative Biotechnology, KU Convergence Science and Technology Institute, Konkuk University, Seoul 05029, Korea

**Keywords:** fish gelatin, marine by-products, nanogel, gelatin methacryloyl, drug delivery system

## Abstract

The gelatin extracted from mammals of porcine and bovine has been prominently used in pharmaceutical, medical, and cosmetic products. However, there have been some concerns for their usage due to religious, social and cultural objections, and animal-to-human infectious disease. Recently, gelatin from marine by-products has received growing attention as an alternative to mammalian gelatin. In this study, we demonstrate the formation of nanogels (NGs) using fish gelatin methacryloyl (GelMA) and their application possibility to the drug delivery system. The fabrication of fish GelMA NGs is carried out by crosslinking through the photopolymerization of the methacryloyl substituent present in the nanoemulsion droplets, followed by purification and redispersion. There were different characteristics depending on the aqueous phase in the emulsion and the type of solvent used in redispersion. The PBS-NGs/D.W., which was prepared using PBS for the aqueous phase and D.W. for the final dispersion solution, had a desirable particle size (<200 nm), low PdI (0.16), and high drug loading efficiency (77%). Spherical NGs particles were observed without aggregation in TEM images. In vitro release tests of doxorubicin (DOX)-GelMA NGs showed the pH-dependent release behavior of DOX. Also, the MTT experiments demonstrated that DOX-GelMA NGs effectively inhibited cell growth, while only GelMA NGs exhibit higher percentages of cell viability. Therefore, the results suggest that fish GelMA NGs have a potential for nano-carrier as fine individual particles without the aggregation and cytotoxicity to deliver small-molecule drugs.

## 1. Introduction

Gelatin, which is produced by partial hydrolysis of collagen, has long been used in a variety of biomedical fields such as pharmaceuticals, medicals, and cosmetics [1,2,3]. In clinics, for instance, gelatin has been used for systemic administration as effective plasma expanders and stabilizers for protein formulations and vaccines [4,5,6]. As a versatile biomaterial, it has also been extensively investigated in the fields of drug delivery systems, possessing numbers of beneficial abilities such as biocompatibility, biodegradability, low antigenicity, and multi-functionality [7,8,9,10]. To date, gelatins originated from porcine and bovine have been mostly used in research as well as industrial purposes, however, religious, social, and cultural objections remain as an ongoing concern alongside generic health problems [11]. For example, the consumption of porcine and bovine products is prohibited in Hindi and Islamic societies, respectively, and vegetarian population have increased worldwide [11,12]. In addition, a critical concern regarding the possible transmission of pathogenic substances inherent in mammalian tissue-derived gelatin products has also been raised (i.e. bovine spongiform encephalopathy) [13]. 

Recently, gelatin derived from marine by-products (e.g. fish skin or bone) has emerged as an alternative to those from mammalian sources [14,15,16]. Fish gelatin has characteristics similar to porcine gelatin, however, it (especially cold water fish gelatin) has a low gelling point below 10 °C with low viscosity whereas mammalian gelatin would remain as the gel state at room temperature with high viscosity [14,15,16]. These distinct gelling behavior of fish gelatin promote the advancement of injectable technologies for the systemic administration and can improve in vivo release [17]. In addition, fish gelatin has higher emulsion stability than bovine gelatin, so it is well suited for production using the emulsion technology [18]. Most of all, the use of fish gelatin is highly cost-effective and environmentally favorable as the material sources can be easily obtained from various by-products of the fish-processing industry, which otherwise will be treated as wastes [19].

Nanogels (NGs) are crosslinked three-dimensional polymer networks in nano-scale that have features of the hydrogel and nanoparticle at the same time [20]. The nano-sized drug delivery vehicles have shown a number of advantages in systemic administration due to the well-known enhanced permeability and retention (EPR) effect, enhancing the stability of drugs against chemical destruction and enzymatic degradation, and lowering the side effects of drugs [21]. In addition, the porosity of NGs contributes to the high drug-loading capacity compared to other solid type nanoparticles (e.g., gold nanoparticles [22], silica nanoparticles [23]) and the swelling property makes controlled release possible. Especially, NGs fabricated by chemical cross-linking shows enhanced mechanical stability under physiological conditions compared with physically synthesized NGs (e.g., liposome [24], micelle [25]), facilitating wider application for drug delivery [7]. 

In this study, we synthesized NGs using fish gelatin methacryloyl (GelMA) through a simple water-in-oil (*w*/*o*) nanoemulsion method. Then the characteristics of fish GelMA NGs were evaluated as well as its biocompatibility. Doxorubicin (DOX) a model drug was loaded to GelMA NGs demonstrated high drug-loading efficiency and sustained release at acidic conditions. Furthermore, the cytotoxicity test of DOX-GelMA NGs was performed using the NIH3T3 cell. These results suggest that fish GelMA NGs have a potential for nano-carrier as fine individual particles without aggregation and cytotoxicity to deliver small-molecule drugs.

## 2. Results and Discussion

### 2.1. Synthesis of Fish GelMA NGs

Fish GelMA NGs were synthesized by photo-polymerization of fish GelMA in a water-in-oil (*w*/*o*) emulsion (Figures S1 and S2). To control the size, fish GelMA NGs were prepared using four different fabrication methods which used deionized water (D.W.) or phosphate buffered saline (PBS, pH 7.4) as an aqueous phase of the emulsion and final dispersion solution after centrifugation as follows; (1) NGs prepared using D.W. only (D.W.-NGs/D.W.), (2) NGs prepared using PBS for the aqueous phase and D.W. for the dispersion solution (PBS-NGs/D.W.), (3) NGs prepared using PBS only (PBS-NGs/PBS), and (4) NGs prepared PBS only and then treated with ultra-sonication (PBS-NGs/PBS after ultra-sonication) (Table S1).

### 2.2. Characterization of Fish GelMA NGs

**Figure 1:**
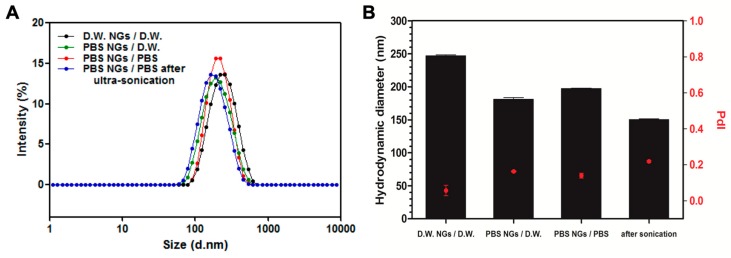
Size distribution of fabricated nanogels (NGs). (**A**) Intensity size distribution by dynamic light scattering (DLS) analysis; (**B**) average hydrodynamic diameter of D.W.-NGs/D.W., PBS-NGs/D.W., PBS-NGs/PBS, and PBS-NGs/PBS after ultra-sonication.

**Figure 2:**
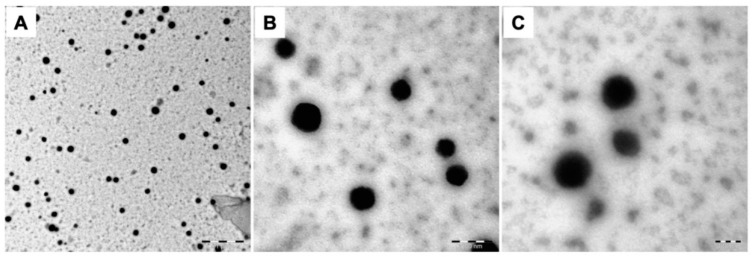
Morphology. Transmission electron microscopy (TEM) images of gelatin methacryloyl nanogels (GelMA NGs). (**A**) Scale bar = 1 μm; (**B**) scale bar = 200 nm; (**C**) scale bar = 100 nm.

The size distribution of nanoparticles is an important factor for the systemic drug delivery agent. Nanoparticles with a size range of 100–200 nm have been reported to reduce uptake by the reticuloendothelial system and increase accumulation in cancer tissues due to the enhanced permeability and retention (EPR) effect [26]. The size distribution of the prepared GelMA NGs was analyzed by dynamic light scattering (DLS). The mean hydrodynamic diameters of D.W.-NGs/D.W., PBS-NGs/D.W., PBS-NGs/PBS, and PBS-NGs/PBS after ultra-sonication were 247.86 nm, 181.13 nm, 197.30 nm, and 150.57 nm with a single narrow peak, respectively (Figure 1A,B). Considering the ideal particle size for systemic drug delivery is 100–200 nm, PBS seems to be a more suitable option for the aqueous phase of emulsion during the synthesis of NGs. The size of PBS-NGs/PBS was slightly larger than that of PBS-NG/D.W., which might be due to the salts contained in the PBS interact with residues of amine and carboxyl groups of GelMA NGs resulting in the restriction of redispersion after centrifugation. However, PBS-NGs/PBS after ultra-sonication showed the smallest size of approximately 150 nm (*p* < 0.001), which is because the agglomeration of PBS-NG/PBS was disentangled by ultra-sonication. All polydispersity index (PdI) measurements of the synthesized GelMA NGs were less than 0.3 regardless of the preparation methods, indicating monodisperse NGs populations. The PdI of PBS-NGs/PBS after ultra-sonication was 0.21, which was significantly higher than that of other NGs (D.W.-NGs/D.W.: 0.05, PBS-NGs/D.W.: 0.16, PBS-NGs/PBS: 0.14) (Figure 1B). This result might be due to the generation of reduced-size particles after the ultrasonic treatment. However, the value was not particularly high, and it can be said that the particles were evenly dispersed. The morphology of the synthesized GelMA NGs was observed using a transmission electron microscope (TEM) (Figure 2). The GelMA NGs had a spherical shape with the size range of 150-200 nm, which was in accordance with that in the DLS analysis. The morphology of GelMA NGs was maintained even after lyophilization. All these results supported the successful formation of the fish GelMA NGs.

### 2.3. Characterization of Drug Loaded Fish GelMA NGs

#### 2.3.1. Drug Loading Efficiency

**Figure 3:**
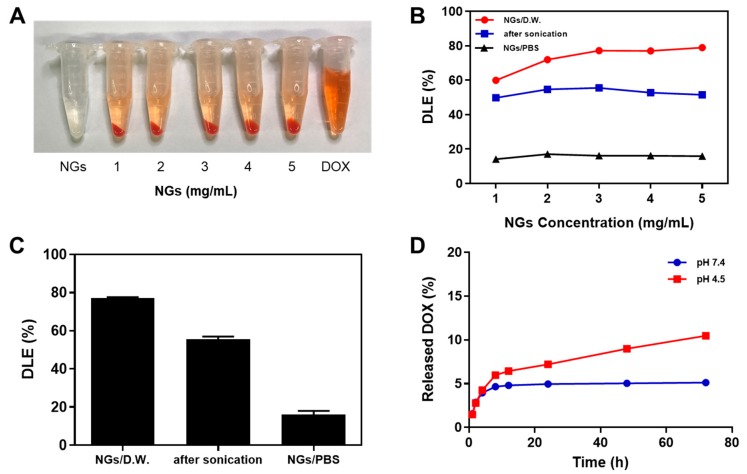
Drug loading efficiency and drug release test. (**A**) Photos of GelMA NGs, DOX-loaded GelMA NGs, and free doxorubicin (DOX) after centrifugation; (**B**) DOX loading efficiency (DLE, %) of GelMA NGs in the concentration range of NGs from 1 mg/mL to 5 mg/mL; (**C**) DLE of GelMA NGs at a concentration of 3 mg/mL. Data are presented as the average ± standard deviation (n = 6); (**D**) in vitro release profiles of DOX from the DOX-GelMA NGs at pH 4.5 and pH 7.4.

Doxorubicin (DOX) is a chemical medication used to treat cancer [27]. DOX was selected as a model drug that can be loaded to fish GelMA NGs by the charge interaction and van der Waals interaction of hydrogen bonding. The drug loading efficiency of GelMA NGs was investigated depending on the preparation method of PBS-NGs/D.W., PBS-NGs/PBS and PBS-NGs/PBS after ultra-sonication in the concentration range of NGs from 1 mg/mL to 5 mg/mL. To load into GelMA NGs, DOX was used at a concentration of 1 mg/mL. The group of D.W.-NGs/D.W. with a size over 200 nm was excluded. Figure 3A shows that, after centrifugation, the DOX loaded GelMA NGs (DOX-GelMA NGs) was precipitated while the unloaded free DOX was in the supernatants. As shown in Figure 3B, the DOX loading efficiencies varied according to the preparation methods of GelMA NGs. The PBS-NGs/D.W. showed the highest loading efficiency at all concentrations, followed by PBS-NGs/PBS after ultra-sonication and PBS-NGs/PBS. The DOX loading efficiency of PBS-NG/D.W. increased from approximately 60% at 1 mg/mL to approximately 80% at 3 mg/mL, while the DOX loading efficiencies of PBS-NGs/PBS and PBS-NGs/PBS after ultra-sonication showed almost constant in the concentration range of 1–5 mg/mL. The average DOX loading efficiencies (%) at 3 mg/mL were 77.2% for PBS-NG/D.W., 52.5% for PBS-NGs/PBS after ultra-sonication, and 16.2% for PBS-NGs/PBS (Figure 3C). The residues of GelMA NGs could be blocked by ions and salts in PBS used as the dispersion solution inhibiting the interaction between DOX and GelMA NGs. Furthermore, DOX (pKa ~ 7.2–8.2) is neutralized at pH 7.4 and has low solubility in PBS due to ions and salts. For these reasons, the DOX loading efficiency of PBS-NG/D.W. is much higher than that of PBS-NG/PBS and PBS-NG/PBS after ultra-sonication. Based on the DOX loading efficiency results, PBS-NGs/D.W. (3 mg/mL) was used to load DOX for further studies (DOX-GelMA NGs).

#### 2.3.2. In Vitro DOX Release Studies

Figure 3D shows the in vitro release profiles of DOX from the DOX-GelMA NGs in buffers with pH 4.5 and pH 7.4 for 72 h. The amounts of DOX released during the first 4 h were approximately 4% in both buffers, showing a similar tendency as an initial-burst release. Up to 72 h thereafter, DOX was slowly and continuously released to approximately 11% in the pH 4.5 buffer, while it was almost not released in the pH 7.4 buffer. These results can be explained by the fact that the amine and carboxyl groups of GelMA NGs and the primary amine group of DOX are both protonated and net positively charged under acidic conditions, reducing the interaction between GelMA NGs and DOX. The enhanced release of DOX at low pH confirmed the potential of DOX-GelMA NGs for the intracellular delivery of cancer drugs. Furthermore, the DOX release can be activated by entering the acidic cancer tissues, reducing side effects on normal tissues [28]. The sustained release behavior of DOX-GelMA NG can reduce the plasma level with low systemic exposure while increasing the duration time of the action [29]. Although only approximately 11% DOX was released at pH 4.5 for 72 h, the drug release rate can be increased by the degradation of GelMA NGs in the cell or in vivo environment [7].

### 2.4. Biocompatibility Test of Fish GelMA NGs

**Figure 4:**
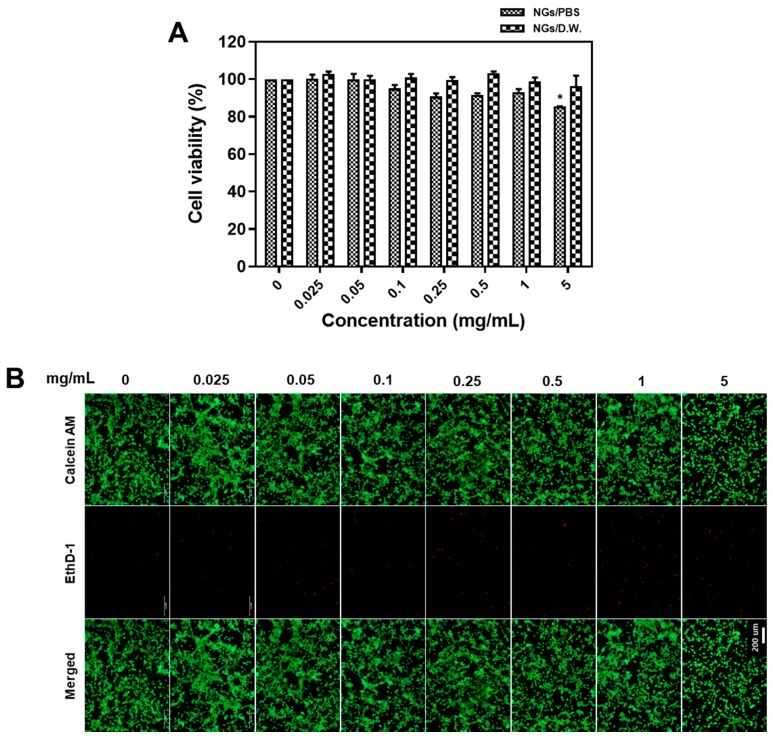
Biocompatibility test. (**A**) Relative cell viability after incubation of NIH3T3 cells with the drug carrier of fish GelMA NGs. Data are presented as the average ± standard deviation (n = 9). (* *p* < 0.05); (**B**) live/dead cell assay after incubation of NIH3T3 cells with the GelMA NGs. Green: Calcein AM, Red: EthD-1. Scale bar = 200 μm.

Cell proliferation test using the MTT assay was carried out using NIH3T3 cells to ensure biocompatibility of GelMA NGs as a drug delivery carrier. The cell viability in the group of PBS-NGs/PBS tended to decrease slightly as the concentration increased, showing a minor cell death at a concentration of 5 mg/mL (*p* < 0.05). On the other hand, the PBS-NGs/D.W. did not show any cell death depending on the concentration up to 5 mg/mL. The unreacted methacryloyl in the GelMA NGs can cause toxic effect and the methacryloyl in PBS NGs-PBS might have been exposed in higher frequency due to increased size by binding with salt in PBS, but both PBS-NGs/D.W. and PBS-NGs/PBS showed high cell survival rates (over 80%) even at high concentration up to 5 mg/mL, suggesting that fish GelMA NGs are biocompatible (Figure 4A). Biocompatibility of GelMA NGs was also assessed by staining cells using a LIVE/DEAD cell assay (Figure 4B). Green fluorescence stained using Calcein AM shows live cells, while red fluorescence stained using EthD-1 shows dead cells. Although cells treated with GelMA NGs of 5 mg/mL showed a slightly reduced proliferation, there was no significant cell death up to 5 mg/mL. These results were in accordance with the results using MTT assays. 

### 2.5. Cytotoxicity Test of DOX-GelMA NGs

**Figure 5:**
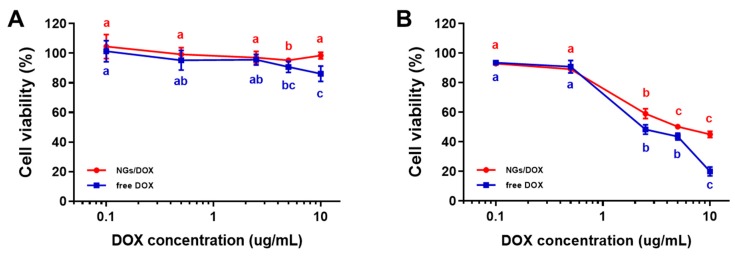
Cytotoxicity test. Cell viabilities of NIH3T3 cells treated with DOX-GelMA NGs and free DOX for (**A**) 4 h and (**B**) 24 h. Data are presented as the average ± standard deviation (n = 9). Cell viability with different letters (a–c) show significant differences depending on DOX concentrations (*p* < 0.05).

The cell growth inhibition of the released DOX from DOX-GelMA NGs was assessed by in vitro cell viability tests in NIH3T3 cells compared with the free DOX. The cells were treated with the DOX-GelMA NGs and free DOX, respectively, for 4 h and 24 h. After incubation for 4 h, the free DOX showed a slight cytotoxicity from 5 µg/mL, while the DOX-GelMA NGs did not show any significant cytotoxicity up to 10 μg/mL. At a DOX concentration of 10 μg/mL, the cytotoxicity of the free DOX was approximately 10% higher than that of DOX-GelMA NGs (Figure 5A). After incubation for 24 h, the free DOX and DOX-GelMA NGs showed significant cytotoxicity from 2.5 µg/mL. At a DOX concentration of 10 μg/mL, cell viabilities were 44.5% for DOX-GelMA NGs and 19.9% for the free DOX (Figure 5B). Although the cytotoxicity of DOX-GelMA NG was 2.23-fold lower than that of the free DOX, it showed effective inhibition of NIH3T3 cell growth after 24 h incubation. These results might be attributed to the sustained release of DOX from DOX-GelMA NGs. Compared with the in vitro DOX release profile described above, where DOX was released to only approximately 11% at pH 4.5 for 72 h, the DOX release from DOX-GelMA NGs appears to be activated in an intracellular environment. Taken together, we confirmed the potential of fish GelMA NGs as a drug delivery carrier. 

## 3. Materials and Methods

### 3.1. Materials

Gelatin from cold water fish skin (Catalog Number: G7041), methacrylic anhydride (MA) and n-octane were purchased from Sigma-Aldrich (St. Louis, MO, USA). Species used for gelatin extraction include cod, pollock, and haddock. The proteins were extracted by boiling skin in water and has been autoclaved at 121 °C for 15–20 min with appreciable hydrolysis. Polyethylene glycol sorbitan monooleate (Tween 80) and Sorbitan monooleate (Span 80) were purchased from Samchun Pure Chemical (Pyeongtaek, Korea). 2-hydroxy-1-(4-(hydroxyethoxy) phenyl)-2-methyl-1-propanone (Irgacure 2959) was purchased from BASF (Minden, Germany) as a photo-initiator (PI) for nanogel polymerization. Doxorubicin hydrochloride (DOX-HCl) was purchased from Tokyo Chemical Industry (Tokyo, Japan). Fetal bovine serum (FBS), penicillin streptomycin (P/S), high glucose Dulbecco’s Modified Eagle’s Medium (DMEM), Dulbecco’s Phosphate Buffered Saline (DPBS), and trypsin-EDTA solutions were purchased from WelGene (Daegu, Korea).

### 3.2. Synthesis of GelMA

GelMA was synthesized by using the method described by Nichol et al. [30] (Figure S1). Briefly, 10% (*w*/*v*) cold water fish gelatin was added to phosphate-buffered saline (PBS, pH 7.4) and stirred at 50 °C until dissolved. Then, methacrylic anhydride (MA) was added at 0.5 mL/min and reacted under stirring at 50 °C for 2 h. The reaction was stopped by dilution with adding an excess amount of PBS. The mixture was then dialyzed against deionized water using the dialysis membrane tubes (Spectra/Por 4, Spectrum Laboratories, Rancho Dominguez, CA, USA) with a molecular weight cut-off (MWCO) of 12–14 kDa for seven days at 40 °C. The final solution was filtered using a solid suspension filtering apparatus (CUOTALAB, Seoul, Korea), lyophilized, and stored at 4 °C until use.

### 3.3. Preparation of GelMA NGs

GelMA NGs were prepared using a water-in-oil (*w*/*o*) emulsion method following the method described by Kim et al. [31] (Figure S2). First, 0.5% (*w*/*w*) PI (Irgacure 2959, BASF) was completely dissolved in D.W. or PBS (pH 7.4) at 80 °C. GelMA was mixed with the PI solution and stirred at 60 °C. Then, the solution was added to the mixture of Span 80 and Tween 80 as surfactants and n-octane as an organic solvent. The certain ratio of organic phase/surfactants/aqueous phase was 66.7:16.7:16.7 according to the result from the previous study. The resulting mixture was homogenized at 8000 rpm for 5 min using a high-speed homogenizer (T 25 digital ULTRA-TURRAX^®^, IKA, Staufen, Germany). After the particles were homogenized, the size of particles was reduced to micro-level. In order to reduce the size to nano, it is further homogenized using an ultra-sonic processor (VC 505, Sonic & Materials, Newtown, CT, USA) under 20 watts. The above process was carried out at 4 °C to prevent the possible temperature rise due to heat energy generated in the homogenization process. The UV light of 250–450 nm was irradiated at 10 W/cm^2^ intensity for 30 min in order to form a gel network of GelMA present in the emulsion. After photo-polymerization, tetrahydrofuran (THF) was added and centrifuged for 10 min (8000 g) to eliminate the organic solvent and the surfactants present in the continuous phase. After the purification process, only the precipitate was re-dispersed in deionized water (D.W.) or PBS (pH 7.4), and then lyophilized to acquire dried nanogel (in powder form).

### 3.4. Preparation of DOX-loaded GelMA NGs

The dried GelMA NGs and doxorubicin (DOX) were completely dissolved in the deionized water, and the pH was adjusted to pH 7.4 by using 0.1 M sodium carbonate (Na_2_CO_3_). The mixed solution was reacted at 4 °C for 24 h. After the reaction, supernatant was removed by centrifugation and the precipitate was collected followed by washing with PBS. Finally, DOX-GelMA NGs were re-dispersed in deionized water, and then lyophilized for further analysis.

### 3.5. Size and PdI Measurements

The hydrodynamic diameter and Polydispersity Index (PdI) of the NGs were measured by Dynamic Light Scattering (DLS) using a nano particle analyzer (Malvern Zetasizer Nano ZS90, Malvern Instruments, Malvern, UK). Each measurement was repeated three times.

### 3.6. Morphology of GelMA Nanogels

The shape of GelMA NGs was observed using a transmission electron microscope (TEM) (JEM-1010, Jeol, Tokyo, Japan). For microscopic observation, 10 μL of the NG solution was loaded on a formvar/carbon coated mesh grid (TED Pella Inc, Redding, CA, USA) and allowed to stand for 2 min. Then the remaining nanogel solution was absorbed using the membrane filter paper. One microliter of 2% phosphotungstic acid solution (Hayashi Pure Chemical Ind. Ltd., Osaka, Japan) was loaded for 1 min, and the remaining staining solution was absorbed using a membrane filter paper. Finally, the grid was washed with deionized water, dried for 12 h and observed under a microscope.

### 3.7. Drug Loading Efficiency

Quantitative determination of the concentration of DOX contained within NGs is accomplished through comparison to a calibration curve of DOX. To obtain the calibration curve, the DOX solution of 500 µg/mL was 2-fold diluted in a series until it was diluted 256-fold, and absorbance was measured at 480 nm using a microplate spectrophotometer (Epoch, BIOTEK, Winooski, VT, USA). The calibration curve was linear over the concentration range. The correlation coefficient was higher than 0.99 for the calibration curve (n = 3). The DOX solution of 1 mg/mL was mixed with the GelMA NG solutions of 1, 2, 3, 4, and 5 mg/mL. The amount of free DOX present in the supernatant after centrifugation was measured using absorbance at a wavelength of 480 nm. The DOX loading efficiency of the NGs was determined by comparing the concentration of added DOX and the unloaded free DOX. The DOX loading efficiency (DLE) (%) was calculated using the following equation.

$$DOX\;loading\;efficiency\left(DLE,\;\%\right)=\left(\frac{D_{0}-D_{1}}{D_{0}}\times 100\right)$$

D_0_: Initial amount of DOX added to the NGs. D_1_: Amount of DOX in supernatant after centrifugation.

### 3.8. In Vitro Drug Release Test

The DOX-GelMA NGs were dissolved in PBS (0.01 M and pH 7.4) and acetate buffer (0.01 M, pH 4.5), respectively, and added to the dialysis membrane tube. The tube was placed in the same dispersion buffer and incubated with stirring of 100 rpm at 37 °C. One milliliter (1 mL) of the sample solution was taken at a fixed time (1, 2, 4, 8, 12, 24, 48, and 72 h). The same buffer was replenished immediately after sampling. The DOX in the collected sample solution was quantitated by measuring absorbance at a wavelength of 480 nm.

### 3.9. In Vitro Cytotoxicity Assay

#### 3.9.1. Cell Culture

For the in vitro cytotoxicity assay, rat fibroblast (NIH3T3) cell line purchased from Korean Cell Line Bank (KCLB, Seoul, Korea) was used. The cell was cultured in Dulbecco’s Modified Eagle Medium (DMEM) supplemented with 10% fetal bovine serum (FBS) and 1% penicillin/streptomycin (P/S) at 37 °C, 5% CO_2_, and 95% O_2_.

#### 3.9.2. MTT Assay

The cell was seeded at the density of 1 × 10^4^ cells/well and cultured for 24 h (96-well plate). Afterwards, NGs were added at various concentrations (0, 0.025, 0.05, 0.1, 0.25, 0.5, 1, and 5 mg/mL) and incubated for one day. Then, 3(4,5-Dimethylthiazol-2-yl)-2,5-diphenyltetrazolium bromide (MTT, Duchefa, Haarlem, The Netherlands) reagent was added with fresh DMEM. After incubation for 2 h, the cells were lysed using 100 μL of dimethyl sulfoxide (DMSO). The absorbance was measured at a wavelength of 570 nm using a microplate spectrophotometer. Cell viability was calculated using the following equation.

$$Cell\;viability\left(\%\right)=\frac{Experimental\;OD_{570}-Blank\;OD_{570}}{Control\;OD_{570}-Blank\;OD_{570}}\times 100$$

The cell survival rate of the free DOX and DOX-GelMA NGs was analyzed using the same method as above. The concentration of DOX was 0, 0.1, 0.5, 2.5, 5, and 10 μg/mL. The MTT assay was performed after 4 and 24 h of drug treatment, respectively, and cell viability was calculated using the above equation.

#### 3.9.3. Live/Dead Cell Assay

The cell was seeded at the density of 2 × 10^4^ cells/well in a 48-well plate and cultured for 24 h. After incubation of 12 h, the cells were incubated with the NGs for 24 h. For survival and apoptotic cell staining, the LIVE/DEAD Viability Kit (Thermo Fisher Scientific, Waltham, MA, USA) was used. The cell was treated with Ethidium homodimer-1 (EthD-1) reagent and Calcein-AM reagent, incubated at room temperature for 45 min, and examined with fluorescence microscope (ECLIPSE Ts2, Nikon, Tokyo, Japan).

### 3.10. Statistical Analysis

Nanogel diameters, PdI data were analyzed using one-way analysis of variance (ANOVA) by different fabrication methods. The MTT cell proliferation assay data were analyzed using a one-way ANOVA by concentrations of NGs and DOX as well as culture time as independent variables. Duncan’s multiple range test was performed to evaluate significant differences among the means. All analysis was performed using SPSS 18.0 (SPSS, Chicago, IL, USA).

## 4. Conclusions

In this study, the suitability of fish gelatin for application to the drug delivery system was investigated. The synthesized GelMA NGs showed different characteristics depending on the preparation method of water-in-oil (*w*/*o*) emulsion. We found that PBS-NGs/D.W., which was prepared using PBS for the aqueous phase of the emulsion and D.W for the final dispersion solution, had a desirable particle size (<200 nm), low PdI (0.16), biocompatibility and high drug loading efficiency (77%). In vitro release tests of DOX-GelMA NGs showed the pH-dependent sustained release behavior of DOX. The anti-tumor effect of the released DOX was confirmed in NIH3T3 cells by the MTT assay. The enhanced release of DOX under acidic conditions can be beneficial for intracellular delivery of drugs. Overall, we were able to confirm the potential of fish GelMA NGs as nano-carriers without aggregation and cytotoxicity to deliver small-molecule drugs. The fish GelMA NG is worth researching as a drug delivery system for a variety of drugs to treat a variety of diseases.

## Supplementary Materials

The following are available online at https://www.mdpi.com/1660-3397/17/4/246/s1, Figure S1: Photo-crosslinking strategy for synthesis of fish gelatin methacryloyl (GelMA) nanogels (NGs), Figure S2: Schematic representation of the procedures to prepare fish GelMA NGs and comparison of NG synthesis parameters, Table S1: Average hydrodynamic diameter and Pdl of D.W.-NGs/D.W., PBS-NGs/D.W., PBS-NGs/PBS, and PBS-NGs/PBS after ultra-sonication.
