# Supplementary material for: Nanogels Derived from Fish Gelatin: Application to Drug Delivery System
Source: Mar Drugs. 2019 Apr 25;17(4):246. doi: 10.3390/md17040246 (PMC6521307; doi:10.3390/md17040246)
Supplement: Supplementary file 1 [file marinedrugs-17-00246-s001.pdf]

Supplementary Materials

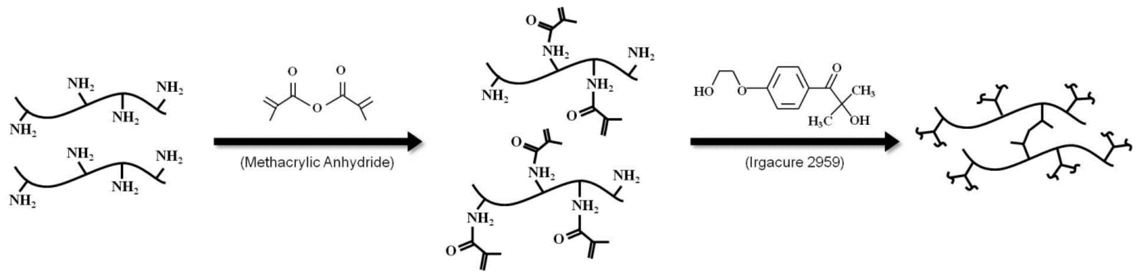

Figure S1. Photo-crosslinking strategy for synthesis of fish gelatin methacryloyl (GelMA) nanogels (NGs). GelMA is synthesized by the reaction of fish gelatin and methacrylic anhydride (MA). The methacryloyl group of MA was conjugated to amine group of the gelatin. GelMA is photo-crosslinkable with photoinitiator (Irgacure 2959) under ultraviolet irradiation (250-450 nm) to polymerize and create gel networks.

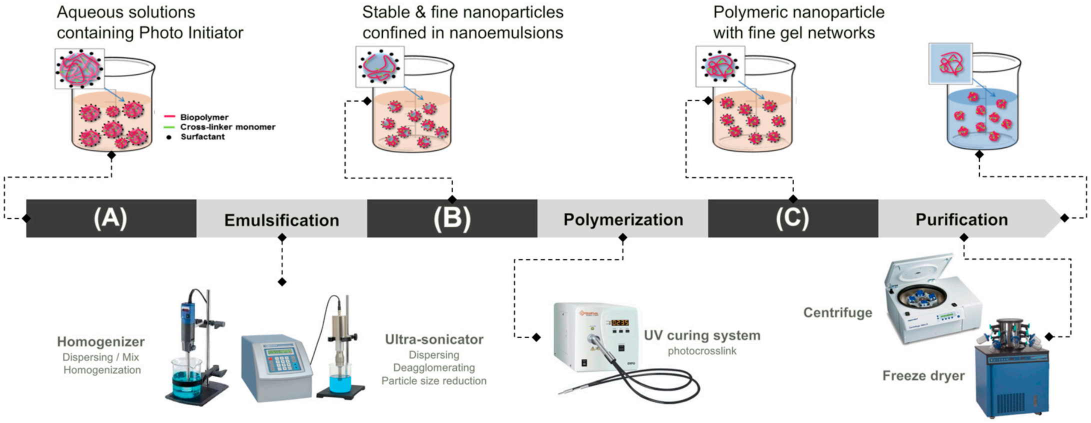

	D.W. NGs / D.W.	PBS NGs / D.W.	PBS NGs / PBS	PBS NGs / PBS after ultra-sonication
• **(A) ~ (C)** : Aqueous solution	Deionized water	PBS	PBS	PBS
• **Purification** : Dispersion solution	Deionized water	Deionized water	PBS	PBS
• **After purification** Additional sonication	X	X	X	O

Figure S2. Schematic representation of the procedures to prepare fish GelMA NGs and comparison of NG synthesis parameters. (A) The aqueous solutions containing fish GelMA and PI were homogenized with organic phase and surfactants to form stable polymeric nanoparticles confined in water-in-oil nanoemulsions. (B) The methacryloyl substituent groups of polymeric nanoparticles were photo-crosslinked under UV light to create gel networks. (C) The NGs were purified and dispersed in PBS or deionized water (D.W.). Figure partly reproduced from Kim et al. with permission from Springer Nature [31].

Table 1. Average hydrodynamic diameter and PdI of D.W.-NGs/D.W., PBS-NGs/D.W., PBS-NGs/PBS, and PBS-NGs/PBS after ultra-sonication. Data are presented as the average ± standard deviation (n=3). Data labeled with different superscript letters (a-d) show significant differences depending on different solvents (p<0.05).

	**Size (nm)**	**PdI**
D.W. nanogels / D.W.	246.86 ± 2.70^{a}	0.05 ± 0.05^{c}
PBS nanogels / D.W.	181.13 ± 4.39^{c}	0.16 ± 0.003^{b}
PBS nanogels / PBS	197.30 ± 0.96^{b}	0.14 ± 0.02^{b}
PBS nanogels / PBS after ultra-sonication	150.57 ± 1.79^{d}	0.21 ± 0.009^{a}
